# Longitudinal trajectory patterns of plasma albumin and C-reactive protein levels around diagnosis, relapse, bacteraemia, and death of acute myeloid leukaemia patients

**DOI:** 10.1186/s12885-020-06754-z

**Published:** 2020-03-24

**Authors:** Kim Oren Gradel, Pedro Póvoa, Olav Sivertsen Garvik, Pernille Just Vinholt, Stig Lønberg Nielsen, Thøger Gorm Jensen, Ming Chen, Ram Benny Dessau, Jens Kjølseth Møller, John Eugenio Coia, Pernille Sanberg Ljungdalh, Annmarie Touborg Lassen, Henrik Frederiksen

**Affiliations:** 1 Center for Clinical Epidemiology, Odense University Hospital, and Research Unit of Clinical Epidemiology, Department of Clinical Research, University of Southern Denmark, Kløvervænget 30, Entrance 216, ground floor, 5000 Odense C, Denmark; 2 OPEN – Odense Patient Data Exploratory Network, Odense University Hospital, J.B. Winsløws Vej 9 A, 5000 Odense C, Denmark; 3 The Polyvalent Intensive Care Unit, Hospital de São Francisco Xavier, CHLO, Estrada do Forte do Alto do Duque, 1449-005 Lisbon, and NOVA Medical School, CEDOC, New University of Lisbon, Campo dos Mártires da Pátria, 1169-056 Lisbon, Portugal; 4 Department of Clinical Biochemistry and Pharmacology, Odense University Hospital, Sdr. Boulevard 29, entrance 40, 5000 Odense C, Denmark; 5 Department of Infectious Diseases, Odense University Hospital, J.B. Winsløws Vej 4, 5000 Odense, Denmark; 6 Department of Clinical Microbiology, Odense University Hospital, J.B.Winsløws Vej 21, 2nd floor, 5000 Odense C, Denmark; 7 Department of Clinical Microbiology, Hospital of Southern Jutland, Sydvang 1, 6400 Sønderborg, Denmark; 8 Department of Clinical Microbiology, Slagelse Hospital, Ingemannsvej 46, 4200 Slagelse, Denmark; 9 Department of Clinical Microbiology, Hospital Lillebaelt, Beriderbakken 4, 7100 Vejle, Denmark; 10 Department of Clinical Microbiology, Hospital of South West Jutland, Finsensgade 35, 6700 Esbjerg, Denmark; 11 Department of Regional Health Research, University of Southern Denmark, 5000 Odense C, Denmark; 12 Department of Emergency Medicine, Odense University Hospital, Kløvervænget 25, entrance 63-65, 5000 Odense C, Denmark; 13 Department of Haematology, Odense University Hospital, and Research Unit of Haematology, Department of Clinical Research, University of Southern Denmark, Kløvervænget 6, entrance 93, 12th floor, 5000 Odense C, Denmark

**Keywords:** Acute myeloid leukaemia, Plasma albumin, C-reactive protein, Infection, Inflammation

## Abstract

**Background:**

No study has evaluated C-reactive protein (CRP) and plasma albumin (PA) levels longitudinally in patients with acute myeloid leukaemia (AML).

**Methods:**

We studied defined events in 818 adult patients with AML in relation to 60,209 CRP and PA measures. We investigated correlations between CRP and PA levels and daily CRP and PA levels in relation to AML diagnosis, AML relapse, or bacteraemia (all ±30 days), and death (─30–0 days).

**Results:**

On the AML diagnosis date (D0), CRP levels increased with higher WHO performance score (PS), e.g. patients with PS 3/4 had 68.1 mg/L higher CRP compared to patients with PS 0, adjusted for relevant covariates. On D0, the PA level declined with increasing PS, e.g. PS 3/4 had 7.54 g/L lower adjusted PA compared to PS 0. CRP and PA levels were inversely correlated for the PA interval 25–55 g/L (R = − 0.51, *p* < 10–5), but not for ≤24 g/L (R = 0.01, *p* = 0.57). CRP increases and PA decreases were seen prior to bacteraemia and death, whereas no changes occurred up to AML diagnosis or relapse. CRP increases and PA decreases were also found frequently in individuals, unrelated to a pre-specified event.

**Conclusions:**

PA decrease is an important biomarker for imminent bacteraemia in adult patients with AML.

## Background

The close monitoring of acute myeloid leukaemia (AML) patients in routine care includes an array of biochemical specimens, amongst these C-reactive protein (CRP) and plasma albumin (PA) that are performed repeatedly during the course of AML. Although AML comprises a group of heterogeneous diseases [1, 2], a common feature is the patient’s higher susceptibility to infectious complications, due to AML’s impact on the immune system or to treatments such as chemotherapy or stem cell transplantation [3]. Neutropenia-associated infection is thus the most common cause of death for patients with AML [1]. In real-life situations it may be difficult to assess the impact of inflammation on specific events (e.g. AML diagnosis or treatment). Studies that assess biomarker levels and their changes over time (longitudinal studies) around well-defined events such as diagnosis, treatment, relapse, bacteraemia, or death may help elucidate this.

There is an abundant literature on CRP as a sepsis marker [4] whereas the literature on CRP in haematological cancer patients is much sparser [5]. In leukaemia patients, we have only encountered few longitudinal studies on CRP levels, all with 63 patients or less [6–10].

Numerous studies show that hypoalbuminemia is invariably associated with a worse prognosis for a wide range of diseases [11, 12]. Hypoalbuminemia has traditionally been related to chronic conditions such as liver failure, malnutrition, or protein losing enteropathy [13, 14]. However, reviews [12, 15–18] and studies in critically ill patients [19–26] indicate that PA may be more important as an inflammatory biomarker, probably mainly related to PA extravasation as a result of capillary leakage. To our knowledge, no study has assessed the PA level as a biomarker of infectious episodes or other events in haematological cancer patients.

Longitudinal studies elucidate whether an abnormal level of the biomarker reflects an acute or a chronic ailment. We recently published longitudinal assessment studies of CRP and PA levels before and after community-acquired bacteraemia [27, 28]. Some of the main findings in these studies were the high inverse correlations between CRP and PA levels and changes. For CRP, changes over time are probably more valid as a mortality predictor than a single measurement [29], but whether this also applies to other outcomes than mortality or to PA is unknown.

In this population-based retrospective study we were able to combine clinical, biochemical, microbiological, and vital status data for 818 AML patients with their 60,209 specimens of CRP and PA. Using CRP as a gold standard inflammatory biomarker in these patients with AML, we determined three aims of the study: i) to relate CRP and PA levels to patient characteristics on the day of AML diagnosis; ii) to describe correlations between CRP and PA levels; and iii) to assess whether changes of daily CRP and PA levels were related to diagnosis, treatment, relapse, bacteraemia, and death.

## Methods

### Setting

In Denmark, the public health system is tax-financed and consequently free of charge for the individual patient and the very few private hospitals are not engaged in management of haematological cancer. All adult (≥15 y) patients with AML are treated in highly specialized haematology departments in tertiary hospitals, which have geographically well-defined catchment areas.

### Derivation of the study cohort

All Danish residents have a unique civil registration number used for all health contacts, which enables linkage between registries [30].

The Danish National Acute Leukemia Registry comprises patients with AML from January 2000, with prospectively recorded clinical and patient-related variables [31]. The AML diagnosis was verified when patients were registered in the database and were based on WHO-defined criteria of blast percentage in bone marrow or blood as well as specific cytogenetic and molecular aberrations [32, 33]. The day of diagnosis (D0) was defined as the day of retrieval of the bone marrow biopsy. The database covers 99.6% of all Danish adult patients with AML, with 90–100% positive predictive values and completeness for almost all assessed variables [34]. From this registry, we retrieved all patients with AML who were followed at the Department of Haematology, Odense University Hospital (OUH), diagnosed from January 2000 through 17 May 2017 (last update at data retrieval). This department has the Region of Southern Denmark (~ 1,221,000 residents) [35] as its catchment area.

We linked data from these patients to the following registries: the Danish National Patient Registry (DNPR) [36], the Danish Civil Registration System (DCRS) [30], biochemistry laboratory information systems (Netlab (Medasys S.A., Littau, Switzerland), BCC (www.cgi.dk/da), LABKA [37]), the OUH Patient Administrative System, and the microbiology laboratory information system MADS [38].

From the DNPR we retrieved comorbidity (excluding haematological cancers) from 1977 (first year of DNPR coverage) up to the patient’s AML diagnosis, as categorized by the Charlson Comorbidity Index [39].

We used the DCRS to retrieve the vital status as per 24 November 2017 (alive, dead, disappeared, or emigrated, including dates of the latter three).

From biochemical specimens recorded in the laboratory information systems we retrieved results for CRP and PA from January 2000 through 2017.

All blood cultures (BCs) were submitted to one of the four clinical microbiology departments (OUH, Hospital of Southern Jutland, Hospital Lillebaelt, Hospital of South West Jutland) in the Region of Southern Denmark. We had data on positive BCs covering 2000–2016, though for 2000–2006 we only had data on BCs submitted to OUH. OUH recorded results in the OUH Patient Administrative System until 2005 and in MADS thereafter, whereas the other three clinical microbiology departments used MADS only.

We computed bacteraemic episodes from all positive BCs, using globally defined criteria [40].

### Analyses of CRP and PA levels

CRP was measured with an immune-turbidimetric principle on modular P® (Roche, Mannheim, Germany). PA was measured on modular P® (Roche) by use of a bromocresol green dye-binding method. All specimen dates refer to date of draw of blood specimens.

### Statistical analyses

**Fig. 1:**
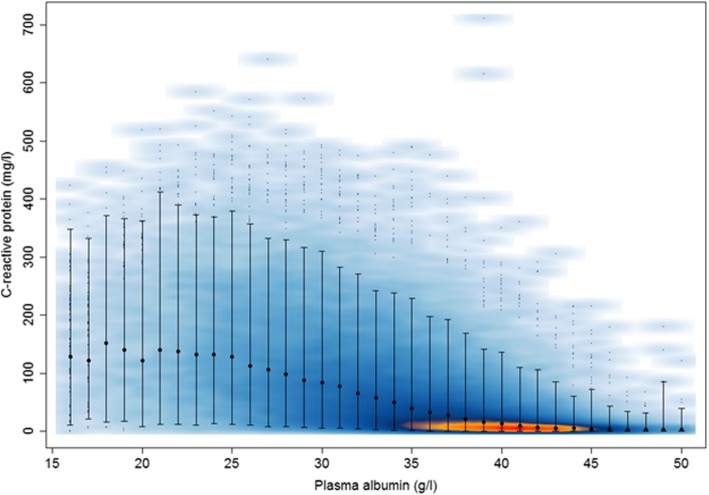
Smoothed scatterplot of C-reactive protein and plasma albumin levels. A smoothed scatter plot of C-reactive protein levels (mg/L) in relation to plasma albumin levels (ranging from 11 to 55 g/L, but 11–15 g/L merged with 16 g/L and 51–55 g/L merged with 50 g/L), based on 818 patients with 60,209 specimens in which both CRP and PA were measured. The smoothed colour displays the density of overplotted points (red - > orange - > dark blue - > light blue represent decreasing density). The medians and 95% ranges of values are shown for each value of PA. 602 (1%) points from areas of lowest regional densities are plotted as small points

The program Stata®, vs. 14, (StataCorp., College Station, TX, USA) was used for all analyses, except Fig. 1 for which R was used [41]. A two-sided *p* < 0.05 was considered statistically significant.

We only included dates of draw of blood specimens on which both CRP and PA were measured. If more than one measurement occurred on the same date we maintained the lowest level of PA and the highest of CRP, thus computing a specimen date as the analytical unit. A number of CRP results were recorded as < 10 mg/L (854/60,209 specimens [1.4%]) or < 5 mg/L (1842 specimens [3.1%]). We therefore randomly re-allocated all CRP levels < 10 mg/L to range from 0 through 9 mg/L, based on the distribution from 10 through 19 mg/L [27]. The same principle was used for CRP levels < 5 mg/L (range 0–4 mg/L, based on the 10–14 mg/L distribution).

Initially, we computed contingency tables of patient characteristics on D0. To assess whether these characteristics were associated with the CRP and PA levels on D0 we performed linear regression analyses, with CRP and PA on D0 as outcomes. We included sex, age group (15–64, 65–80, + 80 y), body mass index (BMI) group in kg/m^2 (< 18.5, 18.5–24.9, 25–29.9, ≥30, unknown), Charlson comorbidity index (0, 1–2, > 2), WHO performance status (PS) (0, 1, 2, 3/4) [42], neutrophil granulocytes in 10^9/L (< 0.5, 0.5–0.9, 1.0–1.4, ≥1.5, not measured), and percent blasts in the bone marrow (0–19, 20–39, 40–59, 60–79, 80–100, not measured) on D0 as independent covariates in crude analyses and in analyses adjusting for the same covariates.

To assess correlations between CRP and PA levels we computed a smoothed scatterplot with CRP for each PA level (integers, ranging from 11 to 55 g/L, but 11–15 g/L merged with 16 g/L and 51–55 g/L merged with 50 g/L due to low numbers) as a separate category. After visual inspection of the scatterplot we computed Pearson’s correlation coefficients for all specimens and separately for 11–24 and 25–55 g/L.

For each patient, a time line was computed, with D0 and dates for the following events assigned a day in relation to D0: first treatment after AML diagnosis, a bacteraemic episode, first AML relapse, and death. For all patients we computed connected line plots with the CRP level in mg/L or the PA level in g/L on the y-axis and the time line (day in relation to D0) on the x-axis, with vertical lines for the above events. We truncated these connected line plots to only include results from 1 year before D0. Because the first treatment after diagnosis was generally very close to D0 we omitted this event in all subsequent analyses.

A clear inverse correlation between CRP and PA levels was observed, both overall and for most of the individual patients. This consistency enabled the merging of results into daily mean CRP and PA levels up to and after the defined events. PA levels were normally distributed, whereas CRP levels were right skewed. In accordance with a previous study [27], longitudinal trajectories did not differ according to whether medians (interquartile ranges) or means (95% confidence intervals [CIs]) were used.

We therefore reported daily mean levels (95% CIs) of PA and CRP, computed from 30 days before through 30 days after the following events: AML diagnosis, first bacteraemic episode after the AML diagnosis, and first AML relapse. For patients who died, we computed daily mean levels (95% CIs) of PA and CRP from 30 days before through date of death.

Because we wished to assess PA and CRP trajectories around the selected events with as little impact as possible from the other events, we made some exclusions. For the AML diagnosis and relapse events, we excluded patients who had a bacteraemic episode within 30 days in relation to this event and death ≤30 days after the event. For the AML diagnosis event we further determined that the first bacteraemic episode should occur > 30 days after the AML diagnosis date. For the bacteraemic episodes, we excluded patients if their bacteraemic episode occurred before the AML diagnosis, ≤30 days after the AML diagnosis, within 30 days before or after the first AML relapse, or ≤ 30 days before death. For death, we excluded patients for whom AML diagnosis, AML relapse, or bacteraemia occurred ≤30 days before death.

Because more severely diseased patients were likely to have more specimens taken, confounding by indication was an important consideration. Hence, to evaluate the robustness of our results we did two things. Firstly, we computed the number of specimens per day in the − 30/30 day interval. Secondly, to assess whether patients with more specimens contributed unequally to the results, we reiterated all plots of mean levels (95% CIs) by only including each patient’s first or last specimen within the day intervals − 30/− 1, 0, and 1/30.

## Results

### Patient characteristics on D0

Table 1 shows patient characteristics on D0. A total of 818 patients were diagnosed with AML between January 2000 and May 2017. Four-hundred and eight patients had 782 bacteraemic episodes, 749 (95.8%) of which occurred on or after D0 and 583 of these (77.8%) occurred ≤1 year after D0 (data not shown). Among the 491 bacteraemic episodes from 2007 through 2016, 452 (92.1%) were detected at the Department of Clinical Microbiology, OUH (data not shown).

**Table 1:** Characteristics^a^ of 818 patients with acute myeloid leukaemia (AML)

Text	Number (%)^**b**^
Females	373 (45.6)
Males	445 (54.4)
Age, years	
Range	15.2–95.6
Median (interquartile range)	69.4 (59.3–76.8)
Charlson comorbidity index	
0	434 (53.1)
1–2	270 (33.0)
> 2	114 (13.9)
No. bacteraemic episodes	
0	410 (50.1)
1	215 (26.3)
2	93 (11.4)
3	61 (7.5)
4	20 (2.4)
5–9	19 (2.3)
Microbiological isolates, first bacteraemic episode	
Mono-microbial Gram-positive	349 (42.7)
*Staphylococcus aureus*	27 (3.5)
Coagulase-negative staphylococci	112 (14.3)
*Streptococcus pneumoniae*	12 (1.5)
Streptococci, other	10 (1.3)
*Enterococcus faecalis*	134 (17.1)
Other	54 (6.9)
Mono-microbial Gram-negative	308 (37.7)
*Escherichia coli*	127 (16.2)
*Klebsiella* spp.	53 (6.8)
*Pseudomonas aeruginosa*	30 (3.8)
Other	98 (12.5)
Mono-microbial fungi	18 (2.3)
Poly-microbial	107 (13.7)
Neutrophil granulocytes (10^9/L)	
< 0.5	182 (22.2)
0.5–0.9	78 (9.5)
1.0–1.4	44 (5.4)
≥ 1.5	215 (26.3)
Not measured	299 (36.6)
Bone marrow biopsy blast percentage	
0–19	14 (1.7)^c^
20–39	283 (34.6)
40–59	182 (22.3)
60–79	154 (18.8)
80–100	132 (16.1)
Not measured	53 (6.5)
Intended treatment at AML diagnosis	
Curative chemotherapy	501 (61.3)
Palliative chemotherapy	91 (11.1)
Best supportive care	218 (26.7)
Unknown	8 (1.0)
Vital status 1 y after the AML diagnosis date	
Deceased	439 (53.7)
Alive	372 (45.5)
Unknown^d^	7 (0.9)
Vital status 2–5 y after the AML diagnosis date	
Deceased	215 (57.8)^e^
Alive	96 (25.8)^e^
Unknown^f^	61 (17.5)^e^

^a^On date of AML diagnosis, except for no. bacteraemic episodes and vital status data

^**b**^Except for “Age, years”, cf. text

^c^These 14 patients had an extra-medullary AML location

^d^Less than 1 year between diagnosis date and latest vital status date (24 November 2017) on which they were alive

^e^Denominator is the 372 patients who were alive 1 year after the AML diagnosis date

^f^Less than 5 years between diagnosis date and latest vital status date (24 November 2017) on which they were alive

### Linear regression analyses for CRP and PA on D0

Results for the 491 patients (60.0%) with both CRP and PA measured on D0 are shown in Table 2, which presents associations between patient characteristics, CRP, and PA at D0.

**Table 2:** Linear regression analyses of C-reactive protein and plasma albumin levels as dependent variables and patient characteristics as explanatory variables on date of diagnosis of acute myeloid leukaemia (AML), based on 491 specimens with both CRP and PA measured

Patient characteristics on date of diagnosis of AML	C-reactive protein		Plasma albumin	
	Crude analysis	Adjusted analysis^a^	Crude analysis	Adjusted analysis^a^
Sex				
Females	1 (reference)	1 (reference)	1 (reference)	1 (reference)
Males	3.80 (−10.5/18.1)^b^	1.78 (− 12.1/15.7)	−1.00 (− 2.10/0.10)	− 1.01 (− 2.02/0.00)
Age group, years				
15–64	1 (reference)	1 (reference)	1 (reference)	1 (reference)
65–80	5.76 (−9.97/21.5)	1.29 (−14.9/17.5)	**−2.01 (− 3.18/−0.84)**^c^	−1.15 (− 2.32/0.02)
+ 80	−3.60 (− 24.1/16.9)	− 16.5 (− 37.6/4.57)	**−4.46 (−5.99/− 2.94)**	**− 2.76 (− 4.28/− 1.24)**
Body mass index (kg/m^2)				
< 18.5	−15.3 (−60.4/29.9)	−19.1 (−63.4/25.1)	−3.32 (−6.78/0.14)	− 2.54 (− 5.73/0.65)
18.5–24.9	1 (reference)	1 (reference)	1 (reference)	1 (reference)
25–29.9	4.12 (− 14.0/22.2)	4.36 (−13.5/22.2)	0.31 (− 1.07/1.70)	0.08 (− 1.21/1.37)
≥ 30	−8.81 (−30.6/13.0)	−6.27 (− 27.6/15.0)	1.33 (− 0.34/3.00)	0.74 (− 0.80/2.28)
Unknown	**30.6 (10.7/50.6)**	17.3 (−2.84/37.4)	**− 2.20 (− 3.72/− 0.67)**	− 0.26 (− 1.71/1.20)
Charlson comorbidity index				
0	1 (reference)	1 (reference)	1 (reference)	1 (reference)
1–2	5.68 (− 10.3/21.7)	5.05 (− 11.2/21.3)	− 1.23 (− 2.46/0.01)	− 0.33 (− 1.50/0.84)
> 2	8.07 (− 13.5/29.6)	−2.91 (− 24.7/18.9)	**−1.89 (− 3.54/− 0.25)**	0.35 (− 1.23/1.92)
WHO performance status				
0	1 (reference)	1 (reference)	1 (reference)	1 (reference)
1	12.4 (− 6.02/30.8)	12.3 (− 6.40/31.0)	**−2.38 (− 3.73/− 1.03)**	**−2.18 (− 3.53/− 0.84)**
2	**36.1 (13.6/58.6)**	**36.7 (13.1/60.3)**	**−4.98 (− 6.63/− 3.34)**	**− 4.21 (− 5.91/− 2.51)**
3/4	**74.9 (51.2/98.6)**	**68.1 (42.2/94.0)**	**−8.41 (− 10.1/− 6.67)**	**− 7.54 (− 9.41/− 5.67)**
Neutrophil granulocytes (10^9/L)				
< 0.5	1 (reference)	1 (reference)	1 (reference)	1 (reference)
0.5–0.9	10.7 (− 13.0/34.5)	5.01 (− 18.0/28.1)	− 0.19 (− 2.00/1.62)	0.65 (−1.02/2.31)
1.0–1.4	15.1 (−14.2/44.5)	1.25 (− 27.2/29.7)	− 0.55(− 2.79/1.68)	0.55 (− 1.51/2.60)
≥ 1.5	**17.9 (0.37/35.5)**	10.1 (−7.22/27.3)	**−2.16 (− 3.50/− 0.82)**	−1.10 (− 2.34/0.15)
Unknown	11.7 (− 11.8/35.2)	1.96 (− 20.9/24.9)	**−2.73 (− 4.52/− 0.94)**	**−2.00 (− 3.65/− 0.34)**
Blast percentage, bone marrow biopsy				
0–19	1 (reference)	1 (reference)	1 (reference)	1 (reference)
20–39	18.1 (− 61.1/97.2)	31.1 (− 46.3/109)	3.71 (−2.48/9.90)	2.49 (− 3.10/8.08)
40–59	21.9 (− 57.8/102)	31.9 (− 46.0/110)	2.91 (− 3.32/9.14)	1.65 (− 3.98/7.28)
60–79	31.0 (− 48.7/111)	40.9 (− 36.9/119)	3.73 (− 2.51/9.96)	2.18 (− 3.43/7.80)
80–100	52.4 (− 27.5/132)	63.0 (− 15.2/141)	3.58 (− 2.66/9.83)	2.05 (− 3.60/7.69)
Unknown	62.1 (−21.2/145)	55.6 (− 25.4/137)	2.28 (− 4.22/8.79)	2.96 (− 2.89/8.81)

^a^Adjusted for all covariates in Table 2

^b^Coefficient (95% confidence interval)

^c^Bold types: statistically significant (*p* < 0.05)

Regardless of significance, covariates with negative coefficients for CRP had positive coefficients for PA and vice versa, except for age groups.

For CRP levels, results were similar in subgroups of sex, age, BMI, Charlson comorbidity index, neutrophil categories, and blast percentage categories, both in crude and adjusted analyses. A clear trend of increasing CRP on D0 was associated with increasing WHO PS, with immaterial differences between the crude and adjusted coefficients. Thus, patients with WHO PS 3/4 had an adjusted level of + 68.1 mg/L CRP in comparison to patients with WHO PS 0.

PA levels were similar in the same subgroups as the CRP levels, except that it declined with increasing age group and WHO PS, with little differences between crude and adjusted analyses. Patients with WHO PS 3/4 had an adjusted PA level of − 7.54 g/L compared to patients with WHO PS 0.

### Correlations between CRP and PA levels

After merging PA levels of 11–15 g/L (*n* = 39) with 16 g/L (*n* = 53) and PA levels of 51–55 g/L (*n* = 47) with 50 g/L (*n* = 49) we computed Fig. 1, which comprises 60,209 specimens. For these 60,209 specimens, the correlation coefficient R was − 0.54 (*p* < 10^− 5^), but as Fig. 1 shows, this inverse correlation was mainly seen in the range 25–55 g/L, for which R was similar (− 0.51, *p* < 10^− 5^, *n* = 56,796). For the remaining 3413 specimens, ranging from 11 through 24 g/L, no correlation was found between the CRP and PA levels (R = 0.01, *p* = 0.57).

### CRP and PA level trajectories for individual patients

**Fig. 2:**
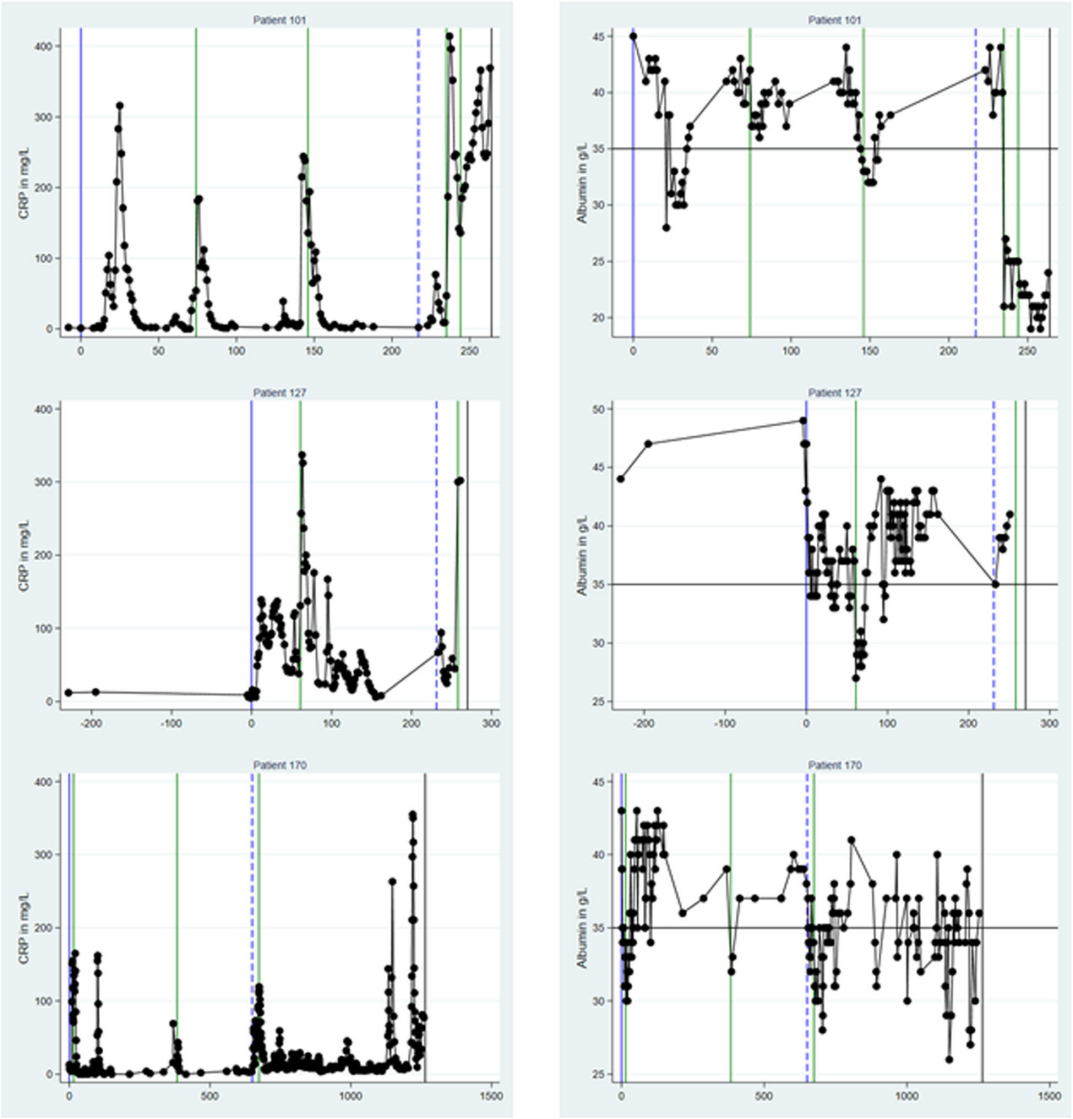
Trajectories of C-reactive protein and plasma albumin levels for three patients. Trajectories of levels of C-reactive protein (CRP) in left column and plasma albumin (PA) in right column for three individual patients (designated patient 101, 127, and 170, using encrypted identification numbers). Date of diagnosis of acute myeloid leukaemia (AML) is designated day 0 (D0) on the x-axis (with a solid vertical blue line) and all other days on the x-axis are depicted in relation to D0. The right-most solid vertical black line shows day of death. In between D0 and date of death, solid vertical green lines show day of diagnosis of a bacteraemic episode and the dashed blue line shows day of relapse of AML. All trajectories exclude specimens retrieved > 365 days before D0. For PA, the horizontal line of 35 g/L shows the threshold between normoalbuminemia (≥35 g/L) and hypoalbuminemia (< 35 g/L)

Fig. 2 shows CRP (left column) and PA level (right column) trajectories for three patients randomly retrieved among patients with the following events: ≥1 bacteraemic episode, AML relapse, and death. All trajectories were truncated to their earliest CRP/PA measurement ≤1 year before D0.

Patient 101 (upper row) had three bacteraemic episodes on D74, D146, and D217 (vertical solid lines), an AML relapse on D217 (vertical dashed line), and died on D264 (vertical solid line on the right). Patient 127 (middle row) had two bacteraemic episodes (D61, D258), an AML relapse on D231, and died on D270. Patient 170 (lower row) had three bacteraemic episodes (D15, D383, D674), an AML relapse on D650, and died on D1264.

CRP levels (left column) generally increased around bacteraemic episodes and prior to death, whereas fewer fluctuations were detected around AML-related events (diagnosis or relapse), although this may be difficult to detect visually for patient 170, because these events were close to two bacteraemic episodes. There were also CRP increases for which we were not able to determine an event that led to this, e.g. patient 101 around D30 and patient 170 around D100 and D1150. CRP was generally close to 0 mg/L in between its fluctuations.

PA (right column) fluctuated inversely to CRP, i.e. when CRP increased, PA declined and vice versa, both related and unrelated to the shown events.

Visual inspection of trajectory patterns for all 818 patients (data not shown) generally showed the same patterns as described for the above three patients. This, together with the high inverse correlations between CRP and PA levels for PA levels ≥25 g/L, enabled the feasibility of computing CRP and PA level trajectories for the aggregated study population.

### Trajectories around main events for aggregated data

Table S1 shows number of patients and specimens used for computing the trajectory curves for the aggregated data in the time span from 30 days before through 30 days after the event (except − 30/0 days for death). Figure S1 shows number of specimens per day within the same time spans.

### CRP level trajectories for aggregated data around diagnosis, relapse, bacteraemia, and death

**Fig. 3:**
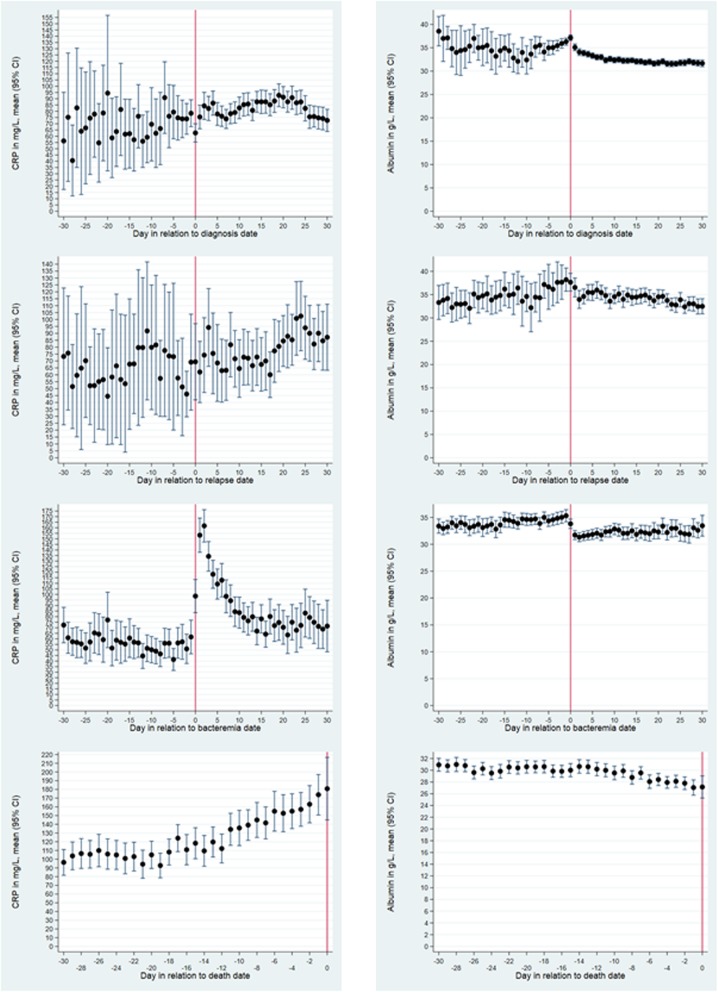
Daily mean levels of C-reactive protein and plasma albumin, aggregated data. Daily mean levels (95% confidence intervals) of C-reactive protein (CRP) in left column and plasma albumin (PA) in right column, in relation to an event (vertical solid line). Events are, from top to bottom: diagnosis of acute myeloid leukaemia (AML), relapse of AML, first bacteraemic episode, and death. Time spans covers from 30 days before to 30 days after the event, except for death that shows 30 days before death. Events occurring earlier than 30 days in relation to another event were excluded

CRP levels showed no clear trend of increases or decreases during the 30 days up to the diagnosis date (Fig. 3, upper row, left side). Most of the mean CRP levels ranged from 55 to 95 mg/L in this period. There were no conspicuous changes in mean CRP levels when AML was diagnosed, but a minor increase started about 7 days thereafter, continuing to day 20 after which it declined again.

For AML relapses, trajectories before these events showed no clear changes (Fig. 3, second upper row, left side) where most of the mean CRP levels ranged from 40 to 80 mg/L. Approximately 15 days after the relapse, CRP levels started to increase, with a peak of ~ 100 mg/L 30 days thereafter.

Mean CRP levels were steadily around 55 mg/L before a first-time bacteraemic episode (Fig. 3, second lower row, left side). On the bacteraemia date, it increased to ~ 100 mg/L, reaching a peak of ~ 160 mg/L 2 days later, after which it declined steadily, reaching a level of ~ 70 mg/L 30 days after the episode.

Mean CRP levels 30–14 days before death were relatively high (100–110 mg/L), but with no clear trend of increases or decreases (Fig. 3, lower row, left side). About 14 days before death, a clear increase commenced, terminating in mean levels of ~ 190 mg/L around death.

### PA level trajectories for aggregated data around diagnosis, relapse, bacteraemia, and death

In the 30 days up to the diagnosis date, mean PA levels fluctuated around 35 g/L, with no clear trend of increases or decreases (Fig. 3, upper row, right side). A decline commenced the day after diagnosis, continuing to a steady level of ~ 31 g/L about 20 days later.

The trajectories around AML relapses (Fig. 3, second upper row, right side) did not deviate materially from those described for the primary AML diagnosis.

Before the first bacteraemic episode, mean PA levels were steadily around 33–34 g/L (Fig. 3, second lower row, right side). It declined on the bacteraemia date, reaching a nadir of ~ 31 g/L 2 days thereafter, after which a slow increase commenced, though pre-bacteraemic levels were not reached 30 days after the bacteraemia date.

Before death, the mean PA levels were steadily around 31 g/L until 14–17 days before, where a decline commenced (Fig. 3, lower row, right side). On the date of death, a mean level of ~ 27 g/L was reached.

### Comparisons between CRP and PA level trajectories for aggregated data

The inverse correlations between CRP and PA levels described above for cross-sectional data (Fig. 1) and for individual patients (Fig. 2) were in the longitudinal data descriptions also seen around bacteraemia and AML relapse, and before death, but were less consistent around AML diagnosis. Treatment data (Table 1) enabled the computation of Figure S2, which shows a decline in PA levels after diagnosis/treatment, both for curative and palliative treatments, whereas no decline was seen if best supportive care was given. For CRP, we computed similar figures in relation to treatment modality, but there were no conspicuous differences between these trajectories (data not shown).

### Reiteration of trajectory plots with each patient’s first or last specimen

The inclusion of each patient’s first or last specimen within each of the three periods day − 30/− 1, day 0, and day 1/30 in relation to the event reduced the numbers of specimens considerably (Table S1). Due to this, many trajectory curves had very wide CIs (data not shown). There were, however, no conspicuous deviations from the trajectories depicted in Fig. 3 (data not shown).

## Discussion

We found high inverse correlations between CRP and PA levels in 818 adult patients with AML. On D0, the linear regression analyses showed minor differences between the univariate and multivariate analyses, which corroborate our results. In cross-sectional analyses of all 60,209 specimens, R was − 0.54 (*p* < 10^− 5^), though a threshold of 24 g/L PA was detected below which no correlation was found. In longitudinal analyses, increasing CRP levels and decreasing PA levels were detected around bacteraemic episodes and prior to death, but also frequently unrelated to events defined beforehand in our study population. In contrast, minor changes in CRP and PA levels were found in relation to AML events (diagnosis or relapse), with the exception that PA levels decreased after diagnosis.

We used the CRP level as a gold standard measure of the magnitude of inflammation. Moreover, a bacteraemic episode is an infection based on well-defined microbial and globally accepted criteria [40]. In order to more closely assess “pure” infection-related, AML-related, and death-related events in the longitudinal analyses we excluded other events types occurring within 30 days.

Our main hypothesis was that PA is an inflammatory biomarker. This was indicated due to its inverse correlation with CRP levels and in relation to its rapid changes over a few days that cannot be explained by a change of the patient’s nutritional status or chronic ailments, also given PA’s long half-life of 20 days [43]. Moreover, PA was not correlated to BMI at diagnosis, which corroborates that it is not a useful biomarker of nutrition [12, 15–18]. Interestingly, the inverse correlations between CRP and PA levels were clearly depicted with increasing WHO PS. WHO PS is, regardless of disease entity, a strong prognostic predictor and further refinement by the incorporation of CRP and PA levels/changes deserves further attention. In recent years, indices based on CRP and PA levels, such as the Glasgow Prognostic Score [44] or the CRP/PA ratio [45], have shown high prognostic predictability for several cancer types. However, the numerous studies focused on solid cancers, they were cross-sectional, and revealed little about possible mechanisms related to the prognostic predictability [44, 45].

Although PA as an inflammatory biomarker was a main finding due to its inverse correlations with CRP levels and changes, other mechanisms were probably also involved in hypoalbuminemia: no correlations between PA and CRP levels were seen for PA < 24 g/L and PA levels decreased shortly after the diagnosis of AML, unparalleled by increasing CRP levels. The latter was explored for treatment subgroups (curative treatment, palliative treatment, best supportive care), indicating that fluid therapy given during treatment could explain this (Figure S2).

Already in 1863, Rudolf Virchow detected leukocytes in neoplastic tissues and thus found a connection between cancer and inflammation [46]. In 1986, Dvorak described tumours as “wounds that do not heal” [47]. During the last two decades, the research field of cancer and inflammation has experienced a renaissance [46, 48, 49]. A review concluded that cancer patients generally had higher CRP levels than controls [50], but as 81 of the 90 studies were cross-sectional we do not know whether the higher CRP levels occurred before the cancer or vice versa.

Smaller studies of longitudinally measured CRP in leukaemia patients (*n* = 20–63) found that CRP levels > 100 mg/L correlated temporally with infectious episodes [6–10]. Some of these studies also assessed CRP levels in leukaemia relapse episodes, which were generally much lower than 100 mg/L [6, 7, 9]. To our knowledge, no study has assessed PA as a biomarker of infectious or cancer episodes in haematological cancer patients, or in any other cancer patient group.

In the present study, the CRP and PA trajectories around the bacteraemic episodes did not deviate from what we have reported for 2472 adult community-acquired bacteraemia patients [27]. This indicates that the pathogenesis related to CRP and PA changes around a bacteraemic episode probably does not differ in relation to the patient group or the degree of immunosuppression. Moreover, much smaller or no CRP and PA changes were detected around AML-related events, which accordingly had little impact on changes around the bacteraemic episodes.

For the AML-related events, the interpretation of the longitudinal CRP and PA trajectories is less straightforward than for the bacteraemic episodes. Firstly, due to low numbers of specimens, caution in interpretation is especially warranted up to the diagnosis and around the relapse. Secondly, although we excluded diagnosis or relapse events for which a bacteraemic episode occurred within 30 days we also found numerous CRP increases and PA decreases that were temporally unrelated to a bacteraemic episode (Fig. 2). This is also found in studies with many non-bacteraemic infectious episodes detected from medical records [6–10]. Thus, other infections than bacteraemia occur close to AML-related events in our study. In spite of these caveats, there were little changes in CRP and PA levels around the AML-related events.

The inverse correlations between CRP and PA levels were also seen in the last 14 days up to death. Even before this 2-week period, mean CRP levels were above 100 mg/L, indicating an ongoing inflammation, and mean PA levels were ~ 30 g/L (i.e. hypoalbuminemia). A recent Swedish study assessed CRP and PA levels up to the death of 155 incurable cancer patients [51]. Though numerous studies have assessed biomarkers of mortality in cancer patients [52], no other study has to their (and our) knowledge assessed CRP and PA levels in the last 2 months before patients’ death. In the Swedish patients, the median CRP and PA levels in the last month before death were 84 mg/L and 23 g/L, respectively. In our study, 654 patients with 5739 CRP/PA specimens in the last month before death had median CRP and PA levels of 108 mg/L and 29 g/L, respectively (data not shown), thus higher CRP and PA levels. Most of the Swedish patients had a broad spectrum of solid tumours, which may in part explain these discrepancies. Unfortunately, the Swedish study did not report longitudinal trajectory patterns, which further hampers comparisons to our study.

In this hypothesis-generating study we have mainly reported descriptive results. We purportedly have not reported discriminatory measures (e.g. sensitivity or positive predictive values). This is mainly due to our definitions, which are not globally defined, and the lack of accurate detection of non-bacteraemic infectious episodes. We had data for all the patients’ hospital admissions as from 1977 and their outpatient visits as from 1995, each with all recorded diagnosis codes [36]. Although the validity of recorded infections may be satisfactory (high positive predictive values as reviewed by [36]) it is dubious whether all infections are recorded in the administrative registries. As only 32.3% of 58,139 bacteraemic episodes were recorded properly in the DNPR [53], milder, and less well-defined, infections were probably even recorded less.

The main strengths of this study are the population-based design (including virtually all adult patients with AML from a geographically well-defined region), complete follow-up, and the high number of patients and specimens. Previous studies on longitudinal CRP analyses in leukaemia patients have included 20–63 patients [6–10] and our study is the first to assess PA. The high number of patients and specimens enabled longitudinal analyses and a two-stage approach in which we first assessed all 818 patients individually and thereafter aggregated their data. Clinically relevant variables of high validity and with few missing values [34] were available at the time of the AML diagnosis. We had valid data on bacteraemic episodes, representing well-defined and globally accepted criteria [40].

Our study also has important limitations. Firstly, most studies that evaluate biomarkers longitudinally are prospective, which enables the retrieval of the same numbers of specimens from all patients, regardless of their current health status. In our retrospective study with its real-life data we expect more specimens from patients with complications, thus confounding by indication may be a concern. This was the main reason for reiterating analyses by the incorporation of each patient’s first or last specimen within pre-specified periods. This did not alter the trajectories materially (data not shown), which is also in accordance with our recent study focusing on this particular aspect [28]. Secondly, non-bacteraemic infectious episodes were not defined and as stipulated above we do not believe this can be done validly from administrative registries, such as the DNPR. Moreover, patients with AML frequently receive prophylactic antibiotics and antimycotics, which will often result in culture-negative microbiological specimens although they clearly have an infection. Thirdly, though the general number of specimens was high, it was low before the AML diagnosis and around AML relapse. Prior to diagnosis of an acute disease, such as AML, it will probably even to a higher degree than described above be the frailer patients from whom specimens are retrieved due to several other reasons than AML per se. Thus, trajectories before the AML diagnosis should be interpreted with extra caution, whereas they are probably more representative for the whole study population in the time period adjacent to and after the diagnosis. Fourthly, we had few clinical variables after the time of diagnosis, of which e.g. BMI and antibiotic/anti-fungal treatment could have been interesting to follow longitudinally. Finally, from 2000 through 2006 we only had microbiological data from OUH, covering 291 bacteraemic episodes. As 452 of the 491 bacteraemic episodes (92.1%) in 2007–2016 were from OUH, we “missed” ~ 30 episodes given the same distribution between OUH and the other three clinical microbiological departments in 2000–2006. Moreover, 10 patients had AML diagnosed in 2017 from which we had no BC results. However, these estimated “missed” 35 bacteraemic episodes (4.5%) will have very little impact on our results.

## Conclusions

The PA level is an important inflammatory biomarker in adult patients with AML. The incorporation of the PA level, together with the CRP level, in mathematically well-founded prediction models deserves further attention. Models that will be able to predict infections or death will be useful for clinicians, both for AML and other immunocompromised patients.

## Supplementary information

**Additional file 1 Table S1:** Numbers of patients and specimens in the aggregated trajectory analyses.

**Additional file 2 Figure S1.** Daily numbers of specimens measuring both C-reactive protein and plasma albumin, from 30 days before through 30 days after diagnosis of acute myeloid leukaemia (AML), relapse of AML, and first-time bacteraemic episode after AML, and from 30 before through date of death. Only comprises specimens in relation to these events if other events occurring ≤30 days were excluded. **Figure S2**. Daily mean levels (95% confidence intervals) of plasma albumin in relation to diagnosis of acute myeloid leukaemia (left column) or treatment (right column), stratified according to curative chemotherapy, palliative chemotherapy, or best supportive care). Time spans cover − 30/30 days in relation to diagnosis/treatment. Events occurring ≤30 days in relation to another event were excluded.

## Data Availability

According to Danish law, national health data cannot be made publicly available. However, analytical schemes in the form of Stata do-files can be reviewed through reasonable request from the corresponding author.

## Abbreviations

AML: Acute myeloid leukaemia
BC: Blood culture
BMI: Body mass index
CI: Confidence interval
CRP: C-reactive protein
D0: Day of diagnosis of acute myeloid leukaemia
DCRS: Danish Civil Registration System
DNPR: Danish National Patient Registry
OUH: Odense University Hospital
PA: Plasma albumin
WHO PS: WHO performance score
