# Supplementary material for: Longitudinal trajectory patterns of plasma albumin and C-reactive protein levels around diagnosis, relapse, bacteraemia, and death of acute myeloid leukaemia patients
Source: BMC Cancer. 2020 Mar 24;20:249. doi: 10.1186/s12885-020-06754-z (PMC7092519; doi:10.1186/s12885-020-06754-z)
Supplement: Supplementary file 1 — Additional file 1 Table S1: Numbers of patients and specimens in the aggregated trajectory analyses. [file 12885_2020_6754_MOESM1_ESM.docx]

**Table S1: Numbers of patients and specimens in the aggregated trajectory analyses**

| **Event** | **Number of patients** | **Number of specimens^1^** | |
| --- | --- | --- | --- |
|  |  | **All specimens** | **First or last specimen^2^** |
| AML diagnosis^3, 4^ | 634 | 9999 | 1422 |
| AML treatment^3^ | 433 | 9226 | 1109 |
| AML relapse^3^ | 126 | 1759 | 238 |
| First-time bacteraemia^5^ | 237 | 5652 | 626 |
| Death^6^ | 341 | 3131 | 384 |

AML = acute myeloid leukaemia

^1^ From 30 days before through 30 days after the event, only including specimens with both C-reactive protein and plasma albumin measured

^2^ Within day −30/−1, day 0, or day 1/30 in relation to the event

^3^ For events occurring >30 days before or after a bacteraemic episode and death occurring >30 days after the event

^4^ For first-time bacteraemic episodes occurring >30 days after the diagnosis date

^5^ For first-time bacteraemic episodes occurring >30 days after diagnosis and >30 days before or after treatment or relapse and death occurring >30 days after the episode

^6^ For death occurring >30 days after diagnosis, relapse, or a bacteraemic episode
