# Supplementary figures and images for: Longitudinal trajectory patterns of plasma albumin and C-reactive protein levels around diagnosis, relapse, bacteraemia, and death of acute myeloid leukaemia patients
Source: BMC Cancer. 2020 Mar 24;20:249. doi: 10.1186/s12885-020-06754-z (PMC7092519; doi:10.1186/s12885-020-06754-z)

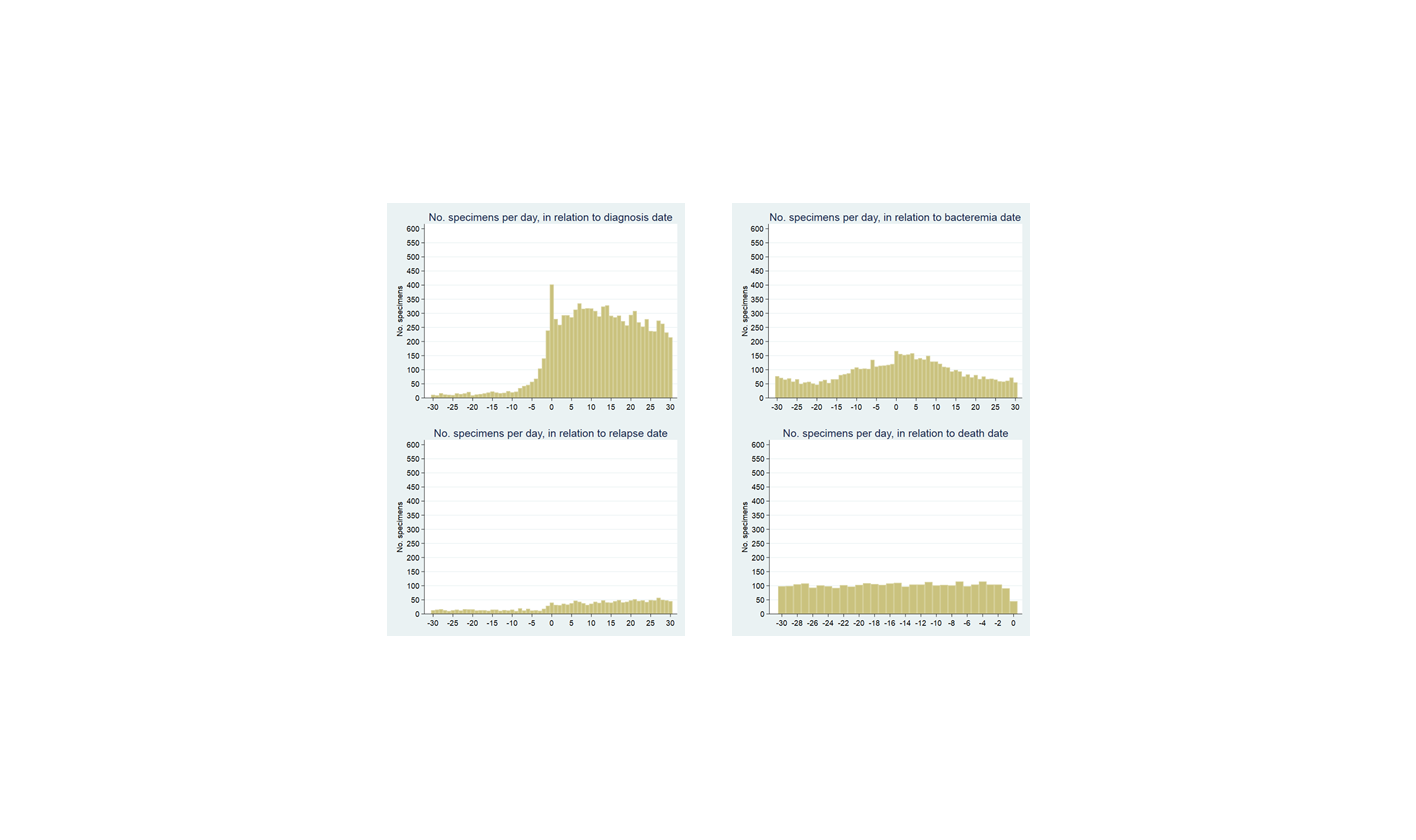

Supplement: Supplementary file 2 — Additional file 2 Figure S1. Daily numbers of specimens measuring both C-reactive protein and plasma albumin, from 30 days before through 30 days after diagnosis of acute myeloid leukaemia (AML), relapse of AML, and first-time bacteraemic episode after AML, and from 30 before through date of death. Only comprises specimens in relation to these events if other events occurring ≤30 days were excluded. Figure S2. Daily mean levels (95% confidence intervals) of plasma albumin in relation to diagnosis of acute myeloid leukaemia (left column) or treatment (right column), stratified according to curative chemotherapy, palliative chemotherapy, or best supportive care). Time spans cover − 30/30 days in relation to diagnosis/treatment. Events occurring ≤30 days in relation to another event were excluded. [file 12885_2020_6754_MOESM2_ESM.zip › figure s1R2.tiff]

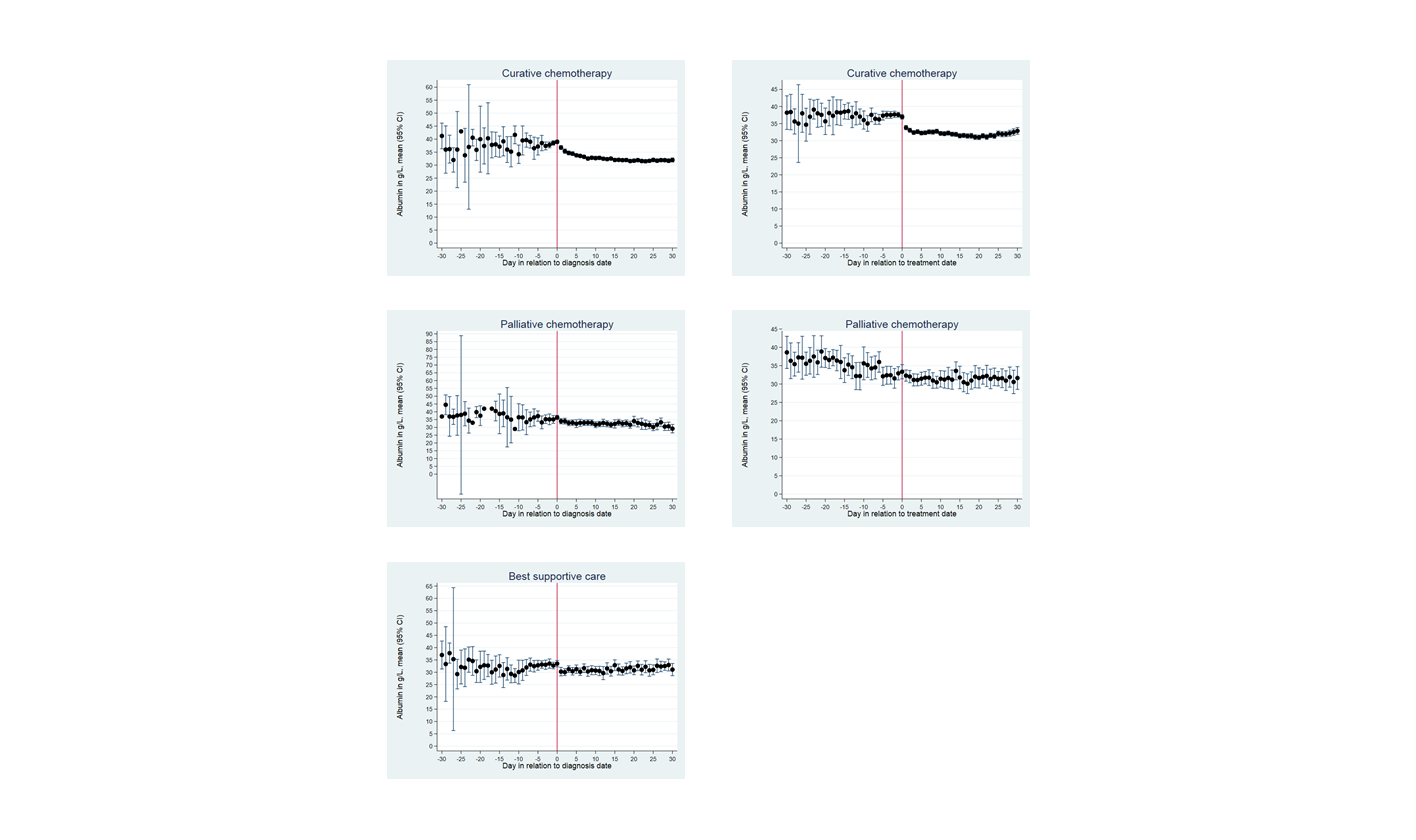

Supplement: Supplementary file 2 — Additional file 2 Figure S1. Daily numbers of specimens measuring both C-reactive protein and plasma albumin, from 30 days before through 30 days after diagnosis of acute myeloid leukaemia (AML), relapse of AML, and first-time bacteraemic episode after AML, and from 30 before through date of death. Only comprises specimens in relation to these events if other events occurring ≤30 days were excluded. Figure S2. Daily mean levels (95% confidence intervals) of plasma albumin in relation to diagnosis of acute myeloid leukaemia (left column) or treatment (right column), stratified according to curative chemotherapy, palliative chemotherapy, or best supportive care). Time spans cover − 30/30 days in relation to diagnosis/treatment. Events occurring ≤30 days in relation to another event were excluded. [file 12885_2020_6754_MOESM2_ESM.zip › figure s2R2.tiff]
